# Smart Fall Detection Framework Using Hybridized Video and Ultrasonic Sensors

**DOI:** 10.3390/mi12050508

**Published:** 2021-05-01

**Authors:** Feng-Shuo Hsu, Tang-Chen Chang, Zi-Jun Su, Shin-Jhe Huang, Chien-Chang Chen

**Affiliations:** 1 Department of Psychiatry, Taichung Tzu Chi Hospital, Buddhist Tzu Chi Medical Foundation, Taichung 42743, Taiwan; ryojames@gmail.com; 2 Bio-Microsystems Integration Laboratory, Department of Biomedical Sciences and Engineering, National Central University, Taoyuan City 32001, Taiwan; s210105@shsh.tw (T.-C.C.); roger54558588@g.ncu.edu.tw (Z.-J.S.); handsome410115@yahoo.com.tw (S.-J.H.); 3 Multimedia Information System Laboratory, Department of Computer Science, National Tsing Hua University, Hsinchu 300044, Taiwan; 4 Chronic Disease Research Center, National Central University, Taoyuan City 32001, Taiwan

**Keywords:** data density functional theory, fall detection, machine learning, posture detection, sensor fusion, ultrasonic sensors

## Abstract

Fall accidents can cause severe impacts on the physical health and the quality of life of those who suffer limb diseases or injuries, the elderly, and their caregivers. Moreover, the later the accident is discovered, the lower the chance of recovery of the injured one. In order to detect accidents earlier, we propose a data-driven human fall detection framework. By combining the sensing mechanism of a commercialized webcam and an ultrasonic sensor array, we develop a probability model for automatic human fall monitoring. The webcam and ultrasonic array respectively collect the transverse and longitudinal time-series signals from a moving subject, and then these signals are assembled as a three-dimensional (3D) movement trajectory map. We also use two different detection-tracking algorithms for recognizing the tracked subjects. The mean height of the subjects is 164.2 ± 12 cm. Based on the data density functional theory (DDFT), we use the 3D motion data to estimate the cluster numbers and their cluster boundaries. We also employ the Gaussian mixture model as the DDFT kernel. Then, we utilize those features to build a probabilistic model of human falling. The model visually exhibits three possible states of human motions: normal motion, transition, and falling. The acceptable detection accuracy and the small model size reveals the feasibility of the proposed hybridized platform. The time from starting the alarm to an actual fall is on average about 0.7 s in our platform. The proposed sensing mechanisms offer 90% accuracy, 90% sensitivity, and 95% precision in the data validation. Then these vital results validate that the proposed framework has comparable performance to the contemporary methods.

## 1. Introduction

The prediction of human falling is an open problem for safety and health issues. For the elderly, unexpected falls often result in dangerous consequences, such as muscle injuries, fractures, internal bleeding, or meningeal hemorrhage. Falls also occur in subjects with dizziness caused by sedation or pain relief after taking medicine. According to a research report from WHO [1], the chance of falls increases with age. The population aged over 65 years old was higher than 15% in many developed countries in 2014, and is expected to exceed 20% by 2050 [2]. In other words, stepping into an aging population is a global phenomenon. The elderly over 65 years old have a 28% to 35% chance of falling each year, and they have an annual increase of 32% to 42% when over 70 years old [1]. The only way to completely prevent fall accidents is to reduce personal activities in daily life. However, this is infeasible. A compromised approach to minimize the risk of falls is to develop a sensing mechanism with the ability to detect, track, and label falling signals.

Contemporary methods for fall detection include two main categorical sensing mechanisms. Wearable-device-based sensing mechanisms often rely on pure accelerometers [3,4], a hybrid of accelerometers and gyroscopes [5,6,7,8,9,10], or personal smartphones [11,12]. The merits of adopting these wearable devices are the low-cost hardware [13,14], the simplicity of use, and the stable sensing mechanism [15]. The raw time-series signals can be mathematically remapped to the three-dimensional features of human motions. Thus, it is easy to estimate the centroid changes of human motion in Euclidean coordinates by utilizing wearable devices. However, the utilization of wearable devices also has intrinsic limitations in daily life usages. Hardware maintenance, inconvenient put on and take off, and the discomfort of setups are issues for elderly users [4,15]. Thus, video-based sensing mechanisms are gradually becoming the mainstream techniques for fall detection [5,16,17]. Among these sensing techniques, human skeleton-based models [17,18,19,20] provide a good performance in detecting and tracking individual human bodies in public spaces. The information of field depth collected from the Microsoft Kinect camera also benefits in the training procedures of skeletal structures [18,20,21,22,23] based on a convolutional neural network (CNN) framework. On the other hand, both the detection and tracking of people in complex reality scenes are challenging problems. For visualizing fall detection, state-of-the-art methods such as real-time compressive tracking [24], fast tracking-detection interaction algorithms [23], tracking-learning-detection algorithms [25], and so forth realize the desired requirement of high-speed human body detection. Machine-learning-based techniques also benefit the development of fast people detection and tracking. A Bayesian-based model using a discriminative correlation filter (DCF) offers an avenue for high-speed human body filtering [26]. A support vector machine (SVM) classifier with the OpenCV library achieved high sensitivity and specificity tasks in [20]. A hierarchical Gaussian process latent variable model realized the detection and tracking of multiple human bodies with reoccurring occlusions in image sequences in [23].

However, video-based detection methods also suffer problems when the tracked subject engages in rapid motions or drastically changes its body shape. In these circumstances, the conventional bounding box techniques deal poorly with the extraction of moving features without any additional technical assistance, even in detecting and tracking the subject continuously [4,15]. To resolve this predicament, we propose a new framework for fall detection by hybridizing the unique sensing mechanisms of video and ultrasonic sensors. We adopt the technique of cascade face detection [27,28,29] to detect the potential tracked subject and a channel and spatial reliability tracking (CSRT) tracker [30,31] for continuous subject tracking. After confirming the tracked subject, the hybridized sensing module collects the longitudinal and transverse motional trajectories from the moving subject. The collected raw data can remap to a three-dimensional (3D) movement trajectory of the moving subject, and then the map is used to estimate the corresponding 3D position and speed centroids. Meanwhile, we also use data density functional theory (DDFT) [4,32] to measure the data cluster number and the corresponding cluster boundaries from the raw data. Finally, we construct a probabilistic map for human fall prediction by considering the 3D centroids and cluster boundaries as prior information. In summary, this research aims to develop a low-cost strategy by hybridizing the sensing mechanisms from a webcam and ultrasonic sensors. The sensing mechanisms and the corresponding detection-tracking algorithms can be built into an MCU to perform human fall detection with acceptable computational complexity.

## 2. Materials and Methods

### 2.1. The Hybridized Platform

**Figure 1:**
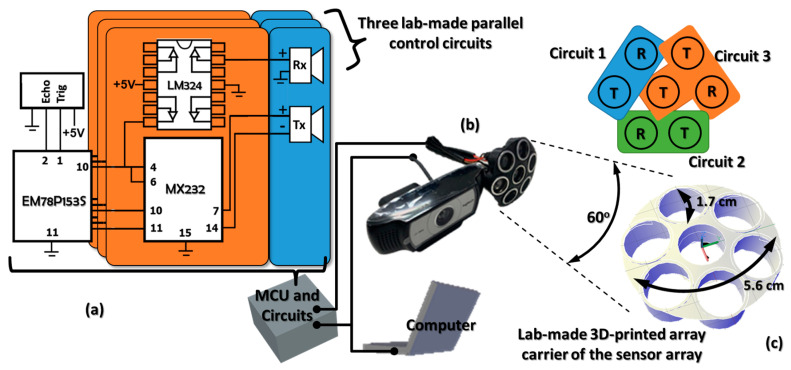
The structure of the proposed hybridized platform includes (**a**) the control and readout circuits of the ultrasonic sensors, (**b**) the hybridized ultrasonic and image sensors, and (**c**) the 3D-printed package of the ultrasonic sensor array. The arrangement of the ultrasonic transmitters (Tx) and receivers (Rx) is also shown in (**c**). The central transmitter has a common control scheme with one of the readout circuits.

To reconstruct a 3D movement trajectory of a tracked subject, we combined the unique sensing mechanisms of an array of highly directional ultrasonic sensors and a commercialized webcam. The sensor array collected the longitudinal time-series signals of the moving subject, while the webcam captured the transverse signals from the tracked bounding box. Figure 1 illustrates the detailed structure of this hybridized platform. There were three lab-made parallel circuits to control and read out the ultrasonic signals, and the tracking and detection of subjects relied on the webcam and computer. We used an 8-bit MCU (Microcontroller unit, EM78P153S, ELAN Microelectronics, Hsinchu, Taiwan) for the general algorithmic computation and control transmission of analog signals. The MCU assigned commands to the MX232 chip (Maxim Integrated, San Jose, CA, USA) for signal emission from the ultrasonic transmitters. Then, the LM324 chip (NXP Semiconductors, Eindhoven, Netherlands) assigned the ultrasonic receivers to assess the ultrasonic responses and passed the received signals back to the MCU. Eventually, the MCU integrated those signals for the algorithmic procedures. To extend the sensing region of the sensor array, we propose a lab-made 3D-printed array carrier. The inclination angle between each sensor was about 15 degrees, and thus the total sensing angle of the sensor array was about 60 degrees. The effective detection range was from 4 cm to 5 m. In the experiments, we aligned the fields of view of these sensors, and thus they had corresponding specific targets. The webcam was aimed at the subject’s face, and the ultrasonic sensors at their chest. To avoid missing packages of ultrasonic signals, we used three independent sets of ultrasonic transmitters and receivers. In the proposed framework, those three receivers respectively returned three sets of distance values to the MCU. If one or more of the returned values indicated that the subject was moving toward the sensing module, these sets of values became principal signals in the longitudinal direction. The built-in algorithm in the MCU then estimated the movement speeds of the subject using those distance values. In the experiments, we collected those signals to construct the 3D movement trajectory map and to train the corresponding probabilistic models.

### 2.2. Tracking Algorithms and Probabilistic Modeling

To precisely detect and track the potential subject, we first combined two different detection-tracking algorithms for recognizing the tracked subject. We used the Haar feature-based cascade classifier [27,28,29] to search the faces of potential subjects. The merit of the cascade classifier is its ability to quickly distinguish targets. Thus, this CNN-based algorithm is quite suitable to conduct the initial search of human faces. Since we assumed the motions of a tracked face could represent whole human motions, we used the coordinates of its corresponding bounding box to estimate the human centroid. In other words, when the cascade classifier captures the first face frame from the potential subject, the center coordinates of the corresponding bounding box are recorded and treated as the human centroid. Then we input the human centroid as an initial feature into the CSRT tracker and use the tracker for the following subject tracking. If the tracker loses the tracked subject in the tracking frames, the cascade classifier automatically re-detects the following video frames. When the classifier successfully captures the subject’s face and its corresponding centroid, the relevant information is input into the CSRT tracker for the following continuous subject tracking again. The trajectories of the centroid are then assembled as two-dimensional (2D) transverse motion features. Eventually, these 2D transverse and longitudinal features are gathered and reconstructed as a 3D movement trajectory map of the subject.

Then, we introduce the discrete-type data density functional theory (DDFT) [4,32] to train a 2D probabilistic map of human falling by utilizing the sets of 3D movement trajectory maps. Under the theoretical framework of DDFT, we treat each data point as a physical particle and estimate their clustering behavior by mapping their feature coordinates into a specific energy space. The data kinetic energy density functional in the energy space represents the cluster significance, while the potential energy density functional represents the data similarity. The corresponding Hamiltonian curve indicates the possible cluster numbers by checking the turning point. The Lagrangian density map can define the possible cluster boundaries by finding the balance points between kinetic and potential energy density functionals [4,32,33]. To train the 2D probabilistic map of human falling, we first define the relevant density functionals. The 2D data kinetic energy density functional (KEDF) t[ρ] is:

(1) $$t\left[\rho \right]=2\pi^{2}\cdot \rho \left(r\right).$$

The function ρ(r) represents a data probability density function (PDF) measured using the Gaussian mixture model [32,34]. The parameter r is a position vector of an observed point. Then, the potential energy density functional (PEDF) u[ρ] is:

(2) $$u\left[\rho \right]=\frac{1}{2}\overset{N}{\underset{i=1}{\sum }}\frac{\rho \left(r_{i}^{\prime }\right)}{\|r-r_{i}^{\prime }{\|}_{r\neq r_{i}^{\prime }}}.$$

The factor N is the number of total data points and $r_{i}^{\prime }$ represents the position vector of the ith data point. Thus, the corresponding Hamiltonian and Lagrangian density functionals are respectively

(3) $$H\left[\rho \right]=\gamma^{2}t\left[\rho \right]+\gamma u\left[\rho \right]$$

and

(4) $$L\left[\rho \right]=\gamma^{2}t\left[\rho \right]-\gamma u\left[\rho \right],$$

where γ is an adaptive scaling factor and has the form:

(5) $$\gamma =\frac{1}{2}\frac{\langle u\left[\rho \right]\rangle }{\langle t\left[\rho \right]\rangle }.$$

〈u[ρ]〉 and 〈t[ρ]〉 are the global averages of PEDF and KEDF, respectively. In the following experiments, we employed the mentioned 2D discrete-type DDFT to construct the 2D probabilistic map of human falling. We utilized the possible cluster numbers to define the stages of human motion and the corresponding boundaries to enclose each stage.

### 2.3. Experimental Framework

To simulate the actual monitoring environments of hospitals and nursing homes, we mounted the proposed hybridized platform on the corner ceiling of the corridor. To imitate the small rooms in hospitals, we designed the detection range to be 2 m. The sensing module triggered the detection requirement while an object approaches the module within 2 m. The corresponding detection-tracking range of the proposed algorithms is from 40 cm to 5 m. The sensing mechanism relies on the synergy of ultrasonic sensors and the webcam in the proposed framework. Thus we aligned and set their targets as human chests and faces, respectively.

**Figure 2:**
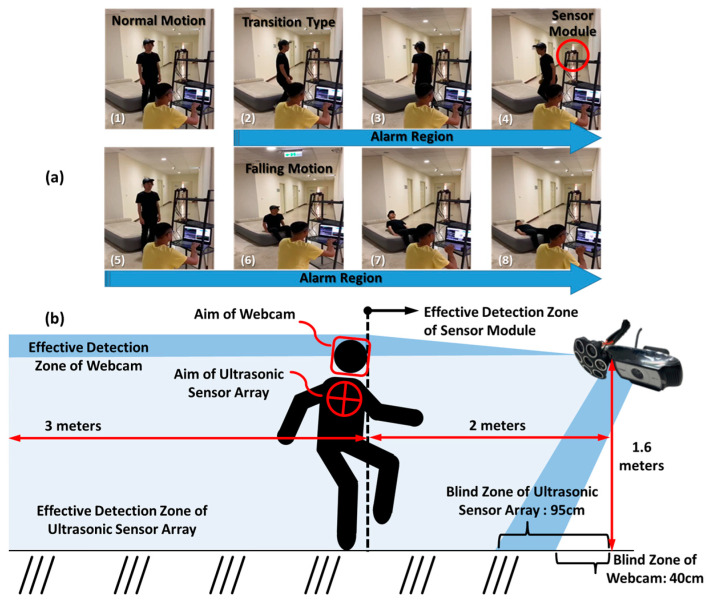
The series of images in (**a**) show the whole proposed experimental environment and falling stages. The sensing module was mounted on a shelf 1.6 m, and the data were collected and returned to the computer for model training. The alarm occurred when the hybridized platform sensed a transition. The image in (**b**) exhibits a detection-tracking scene of the proposed sensing scheme. Critical dimensions of effective and blind zones are clearly delineated. The targets of the ultrasonic sensor array and webcam were human chests and faces, respectively.

Figure 2 illustrates the experimental environment and depicts a sensing scene. The sensor module was mounted on a shelf 1.6 m high to simulate the sensing condition. To simulate dizzy postures, each subject rotated in place for 30 s during each experiment. According to each subject’s feeling of dizziness, they fell in different ways. The hybridized platform simultaneously detected and tracked the target subject during the experiment and returned the 3D data points. We defined the starting point from the end of the rotation to falling and lying down as a fall event. We collected 140 individual fall tests from seven healthy adults as datasets for training the 2D probabilistic map of human falling. The mean height of these subjects was 164.2 ± 12 cm. To avoid detection errors, the algorithm turned off the tracking function five seconds after it detected a fall event automatically. The number of data points collected during an individual test would be slightly more than that during a fall event. Thus, a fall event only included 30 data points, from the 3rd to the 33rd, in order to avoid initial and final detection errors. In the overall 140 individual tests, we collected 4200 data points.

**Figure 3:**
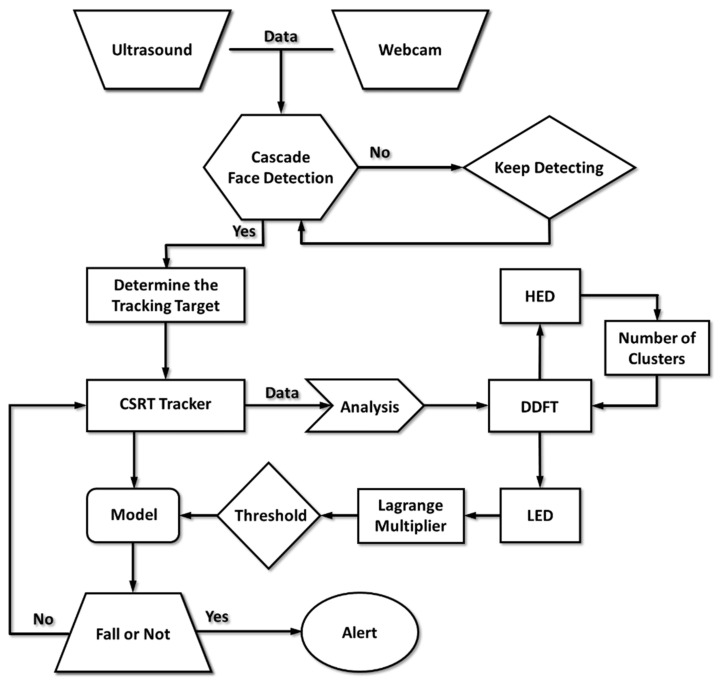
The systematic structure illustrates the stages of data collection, human detection and tracking, probability model training, and the trigger conditions of the fall alarm.

**Figure 4:**
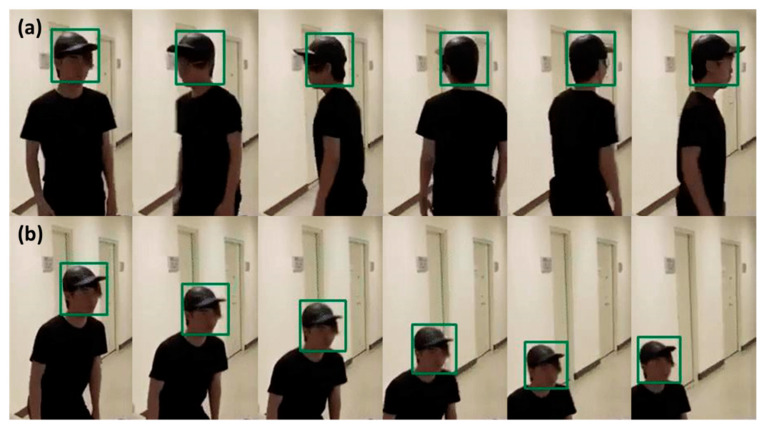
The image series shows the performance of the synergy of cascade face detection and CSRT tracker. The image in (**a**) exhibits the tracking results when the subject stands with their back to the camera. The image in (**b**) shows the tracking results when the subject is in a sudden fall situation.

Since there was a significant dimensional mismatch between the ultrasonic data and image data, we used decorrelated batch and group normalizations to deal with the data collected from different sensors [35,36]. Then, the normalized data points became the input datasets of the discrete-type DDFT for the model training. In the experimental framework, we used 10-fold cross-validation to validate the correctness of the probabilistic map of human falling, in which 10 fall events were randomly selected each time as a testing set of a fall event. Figure 3 illustrates the detailed algorithmic architecture and a complete system operation flow chart. To ensure the detection-tracking algorithm worked when subjects stood with their backs facing the camera, we adopted a two-stage scenario. The synergy of cascade face detection and the CSRT tracker ensures the continuous tracking of human faces. When the subject stands with their back turned toward the camera, the CSRT tracker can still operate well according to the pixel and local information [30,31]. Figure 4 shows the tracking results of the synergy of cascade face detection and CSRT tracker.

The sensing performance in the proposed framework significantly relies on the synergy of ultrasonic signals and webcam images. As shown in Figure 1, the MCU receives the ultrasonic signals and judges whether there is a subject entering the detection zone. Once a subject enters the detection zone, the MCU triggers the cascade face detection algorithm to search the subject’s face. Then, the signals of successful face detection trigger the CSRT tracker for continuous face tracking. The synergy of the cascade face detection algorithm and the CSRT tracker capture the motion signals of the subject and estimate the corresponding transverse centroid motions. Then the ultrasonic sensor array collects the longitudinal signals and estimates the longitudinal centroid motions. These characteristics of centroid motions are then transmitted to the computer to construct a corresponding 3D movement trajectory map. To train the corresponding probabilistic map, we utilized the discrete-type DDFT for statistical learning. We mapped the data of centroid motions into energy spaces, called Hamiltonian and Lagrangian spaces. Under the theoretical framework of the discrete-type DDFT, we estimated the possible cluster numbers and the corresponding cluster boundaries. We used this information to train and build the desired probabilistic map of human falling. In the estimation of the Hamiltonian curve, we used the Gaussian mixture model as the kernel of the data PDF.

## 3. Results

**Figure 5:**
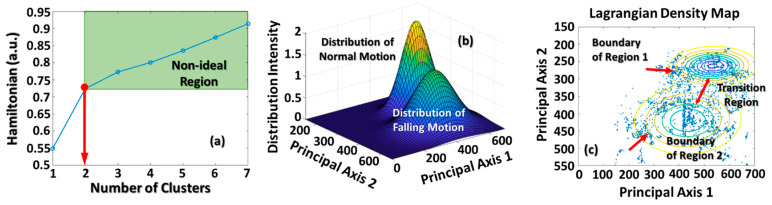
This figure shows (**a**) the Hamiltonian curve, where a red arrow indicates the most probable cluster number, and (**b**) the real data distributions estimated using the discrete DDFT. (**c**) The corresponding cluster boundaries of these two clusters. There is a transition region between clusters, and it represents the transition motions between normal walking and falling.

Figure 5 illustrates the theoretical results of the most probable cluster number and corresponding cluster boundaries estimated by estimating the Hamiltonian curve and the Lagrangian density map, respectively. As shown in Figure 5a, the Hamiltonian curve shows energy fluctuations under different cluster numbers. The slopes of these energy fluctuations gradually became stable as the input cluster number increased above two. This means that the most probable cluster number should be two, as the red arrow indicates. The non-ideal region depicted in Figure 5a exhibits the uncertainty of the collected data. It might be caused by detection errors, an algorithmic flaw, or other data imperfections [4,32]. According to the theoretical cluster number, Figure 5b exhibits the data distribution estimated by employing the discrete-type DDFT. The distribution intensity of the normal motion was higher than that of the falling motion. However, the scattering region of the falling motion was larger than that of the normal motion. Figure 5c also exhibits a similar phenomenon. This reveals that human falling motion has higher instability than that of normal walking. Meanwhile, there are still many data points between these two clusters. These data points indicate that there must be a transition stage that exists between the normal and falling motions. Thus, we defined the regions which are 1.5 standard deviations beyond these cluster distributions with as the transition regions.

**Figure 6:**
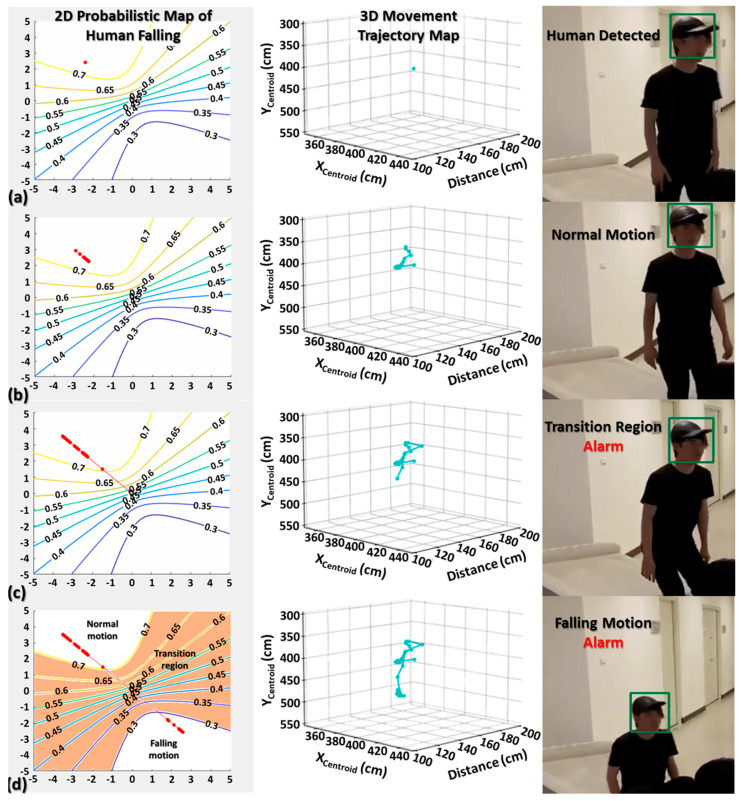
This figure illustrates the corresponding 2D probabilistic maps, 3D movement trajectory maps, and the detection-tracking results of (**a**) detecting a potential subject, (**b**) normal motion, (**c**) transition region, and (**d**) falling motion. Any motions making the data points appear in the transition region might trigger alarms.

To visualize the whole detection-tracking process, Figure 6 illustrates the corresponding 2D probabilistic maps, 3D movement trajectory maps, and the detection-tracking results of each stage. The 2D probabilistic map of Figure 6d illustrates the proposed stages of normal motion, the transition region (the pink region), and falling motion. When the data points move into the transition region, the platform will continuously alarm users. From starting the alarm to an actual human falling was on average about 0.7 s for a sudden situation under the proposed sensing regime. Motions with staggering gaits or significant centroid shifting might also trigger the alarms.

In the experiments, the integrative manifestations of face detection and tracking algorithms steadily reached an accuracy of 98% within the detection range, and the accuracy slightly degraded to 96% when outside of the detection range. The accuracy of the returned ultrasonic signals approached 99% regardless of whether there was a subject inside the detection zone. The sensitivity and precision rates of the human fall alarm were 90% and 95%, respectively. In the model validation procedure, we adopted 10-fold cross-validation. The data points collected by the hybridized sensing module were clustered into four groups. The results classified in the true-positive (TP) and true-negative (TN) groups represent the correct data point classification. The data points belonging to the transition and falling regions were classified into the TP group, whereas those belonging to the normal motions were placed in the TN group. Thus, the number of points in the TP group is much larger than that in the TN group. The points in the false-positive (FP) group are those that should be in TN but were classified into the TP group by the model. The points in the false-negative (FN) group were classified as normal motion but should have been identified as transition/fall. To further compare the performance between the contemporary techniques and the proposed method, we also estimated the corresponding accuracy, sensitivity, and precision. The relevant results and comparison with Kinect-camera-based methods are respectively listed in Table 1 and Table 2. The definitions of these parameters are as follows [37]:

(6) $$\mathrm{Accuracy}=\frac{\mathrm{TP}+\mathrm{TN}}{\mathrm{TP}+\mathrm{TN}+\mathrm{FP}+\mathrm{FN}},$$

(7) $$\mathrm{Sensitivity}=\frac{\mathrm{TP}}{\mathrm{TP}+\mathrm{FN}},$$

(8) $$\mathrm{Precision}=\frac{\mathrm{TP}}{\mathrm{TP}+\mathrm{FP}}.$$

## 4. Discussion

To minimize the module size and reduce the computational complexity, previous studies often used a single sensing mechanism to recognize the changes in human postures. However, this limits the amount and the diversity of information collected. In this article, we proposed a new type of sensor fusion platform combining two heterogeneous sensors. A webcam records the transverse human motion signals, while an ultrasonic array captures the longitudinal motion signals. We collected these features to construct 3D human movement information. The proposed 3D-printed array package improved the detection performance of the ultrasonic sensor array. We also defined the effective detection zone according to the physical characteristics of the employed sensors. To simultaneously reduce the computational complexity and the hardware cost, we successfully recognized the stages of human motion by only employing the centroid changes in 3D space. To improve the detection-tracking algorithms, we combined the techniques of cascade face detection and the CSRT tracker. The synergy of the cascade face detection algorithm and the CSRT tracker ensures the feasibility of continuous face tracking of moving subjects. Even if subjects stands with their backs facing the webcam, the CSRT tracker can identify the subject’s face by checking the pixel and local information.

We also employed discrete-type DDFT to build the probabilistic map of human falling. The cluster numbers and the corresponding cluster boundaries were used to train the corresponding probabilistic model. There were two cluster boundaries in the Lagrangian map in our experiments, indicating that there was a transition region between these two clusters. According to this finding, we concluded that the category of human falling has three stages: normal motion, transition, and falling. We also found that there was a longitudinal motion shift in the 3D movement trajectory map when the subject was approaching the transition region. In Figure 6b–d, there is a longitudinal motion shift along the distance axis. Practically, any data point entering into the transition region might trigger alarms. The time for an alarm to be triggered from the transition region to a sudden fall was about 0.7 s under the proposed framework. The vital results listed in Table 2 validate that the proposed hybridized sensing framework has comparable performance to the contemporary methods.

## 5. Conclusions

Inspired by the sensing mechanisms of the Microsoft Kinect cameras, we proposed a hybridized sensing platform. We respectively utilized webcam and ultrasonic sensors to capture the transverse and longitudinal motion signals from subjects. The longitudinal motion signals can offer the deep features of the subject’s motions. The human skeleton model built from the Kinect cameras has provided fruitful research results in varied applications. However, skeleton model training is a process with high time complexity, and the participants also have to accommodate the high hardware cost. In this research, we proposed a low-cost strategy by hybridizing the sensing mechanisms from a webcam and ultrasonic sensors. To embed the algorithms into an MCU, we had to develop a methodology with low computational complexity. The recognition of the human skeleton under this scheme is a challenging problem. Fortunately, both the human face and body have similar behavior characteristics in normal motion or in a sudden fall, and thus have similar values of movement distance and speed. Thus, we employed the centroid of the tracked face to represent that of the human body.

The proposed algorithms and sensing mechanisms were embedded in an MCU to perform human fall detection. Multi-object detection and continuous tracking are possible if alterations were made to the relevant algorithms and methods. However, the designed sensing mechanisms are only suitable for the detection and tracking of one human since the ultrasonic sensor array can only aim at one human chest at a time. Thus, the proposed framework has this intrinsic limitation. In short, this research provides a low-cost monitoring method. In the future, we hope to improve the accuracy of tracking and apply it to multi-person monitoring and practical applications in hospitals.

## Figures and Tables

**Table 1:** The quantitative results of the proposed sensing system.

True Positive (TP)	True Negative (TN)	False Position (FP)	False Negative (FN)
25,725	12,026	1292	2956

**Table 2:** The comparison between Kinect-camera-based methods and our method.

Methods	Accuracy	Sensitivity	Precision
Ref. [18]	0.99	--	--
Ref. [20]	--	1.00	0.98
Ref. [21]	0.91	0.91	0.91
Ref. [22]	0.72	--	--
Ref. [23]	--	0.92	0.85
This Work	0.90	0.90	0.95

--: Not provided.

## Data Availability

Data sharing not applicable.
